# Separate and overlapping specificities in rheumatoid arthritis antibodies binding to citrulline- and homocitrulline-containing peptides related to type I and II collagen telopeptides

**DOI:** 10.1186/s13075-014-0515-z

**Published:** 2015-01-09

**Authors:** Sanna Turunen, Pekka Hannonen, Marja-Kaisa Koivula, Leila Risteli, Juha Risteli

**Affiliations:** Department of Clinical Chemistry, Institute of Diagnostics, University of Oulu, P.O. Box 5000, Oulu, FI-90014 Finland; Northern Laboratory Centre NordLab and Medical Research Centre, Oulu University Hospital, Oulu, Finland; Department of Medicine, Central Hospital, Jyväskylä, Finland

## Abstract

**Introduction:**

Our objective was to find out if there are antibodies binding to homocitrulline-containing type I and II collagen carboxyterminal telopeptides in sera of patients with rheumatoid arthritis (RA), and if these antibodies cross-react with citrulline and homocitrulline in the same peptide sequence.

**Methods:**

A total of 72 RA and 72 control sera were analyzed for binding using enzyme-linked immunosorbent assay to citrulline- or homocitrulline-containing type I and II collagen carboxyterminal telopeptides, as well as to cyclic citrullinated peptide (CCP) and to mutated citrullinated vimentin (MCV). Specificities of the antibodies were tested using inhibition-ELISA.

**Results:**

Of the RA sera, 39 (54%) and 41 (57%) were positive for binding to CCP and MCV, respectively. Further, 34 (47%) and 30 (42%) of the patients had specific antibodies binding to and being inhibited by citrulline-containing type I collagen telopeptides and by citrulline-containing type II collagen carboxyterminal telopeptides, respectively. The corresponding figures regarding homocitrulline-containing type I and homocitrulline-containing type II collagen telopeptides were 16 (22%) and 14 (19%). Most of the patients, who were seropositive for citrullinated peptides, showed binding in multiple assays. A total of 10 (14%) RA patients were positive for all the tested peptide pairs, while 28 (39%) of them had antibodies that contained overlapping specifities between citrulline and homocitrulline in the same peptide sequence.

**Conclusions:**

Antibodies to both citrulline and homocitrulline containing type I and II collagen telopeptides can be found in sera of RA patients. These antibodies are not constant from one RA patient to another, but contain separate or overlapping specificities within the same peptide sequence varying between individuals. Our results suggest some relationship between citrulline and homocitrulline-recognizing antibodies, since homocitrulline antibodies exist mainly in individuals seropositive to anti-CCP and anti-MCV.

**Electronic supplementary material:**

The online version of this article (doi:10.1186/s13075-014-0515-z) contains supplementary material, which is available to authorized users.

## Introduction

The search for citrullinated antigens in patients with rheumatoid arthritis (RA) has been in focus during recent years. The identification of the original immunogen for their production could be the key to the prevention of the immune response and the consequent cascade of the inflammatory process leading to tissue damage and loss of function. Antibodies against citrullinated proteins are a frequent finding in RA patients and may precede the onset of clinical symptoms by several years [1-3]. We have previously shown the presence of antibodies binding to citrullinated type I and II collagen telopeptides in sera of RA patients [4]. Several tests for antibodies binding to citrullinated peptides have been developed and some are in clinical use; however, they do not present the original antigen but rather rely on the cross-reaction of anti-citrullinated protein antibodies (ACPA). The presence of ACPA in the sera, measured by the anti-cyclic citrullinated protein (anti-CCP) method, has been included as an important component of the American College of Rheumatology – European League against Rheumatism (ACR – EULAR) 2010 classification criteria for RA [5]. Anti-CCP, however, measures the binding of the sera to a mixture of several citrullinated peptides. These peptides have been chosen to give an optimal distinction between controls and patients with RA [6]. Nevertheless, the presence of ACPA in RA patient sera has been connected to poor prognosis and erosive course of the disease.

Lately, also, the contingent effect of carbamylation on the pathogenetic process of RA has become of interest. Carbamylation through myeloperoxidase is part of a natural defense mechanism and takes place in inflammatory conditions (reviewed in [7]), and has been proposed as a link between smoking, uremia, inflammation and atherogenesis [8]. The product of lysine carbamylation is homocitrulline, a structural homolog of citrulline. Recently antibodies against both citrullinated and carbamylated antigens were found to be present simultaneously in sera of RA patients [9]. We have shown that a low amount of homocitrulline can be present in RA tissues simultaneously with citrulline [10]. An interesting coexistence of citrullination and myeloperoxidase activity is found at least in neutrophil extracellular traps (NETs). NETs are also present in periodontitis [11] and by containing citrullinated proteins they may be involved in ACPA formation and trigger subsequent development of RA [12,13].

Altough ACPAs arise in RA patients years before the onset of clinical disease, the origin of these antibodies is not known. The RA patients’ antibody repertoire has been reported to go through epitope spreading between citrullinated antigens [3,14,15]. The specificity of these antibodies for citrulline-containing epitopes is certain. However, the antibodies have been found to be of low avidity [16]. We have demonstrated that antibodies against citrullinated proteins can be developed by immunizing rabbits either with citrullinated or carbamylated (homocitrulline-containing) antigens [17]. Also, antibodies binding to homocitrulline-containing fibrinogen have been detected in RA patients. Furthermore, these were found to cross-react with similar citrulline-containing sequences [18]. In this study, we shed light on the RA patients’ antibody repertoire binding to citrulline- and homocitrulline-containing peptides related to type I and type II collagen carboxyterminal telopeptides. The objective of this study is to define the binding properties of the antibodies binding to citrullinated proteins, not to present another method for RA diagnosis.

## Methods

### Ethical considerations

The study was conducted according to the requirements of the Declaration of Helsinki and was approved by the ethics committee of the Central Hospital of Jyväskylä. All patients gave verbal informed consent before inclusion in the study. In the 1980s, when the study was designed and conducted, no Good Clinical Practice instructions existed. Verbal consent in clinical studies was common praxis. The study design was accepted by the ethics committee of the hospital.

### Subjects and controls

A total of 72 RA patients diagnosed in the years 1987 and 1988 in the Jyväskylä Central Hospital according to the Ropes criteria (Ropes 1959), whose sera were available were included in the present study [19]. At the baseline, the patients were characterized as suffering either from polyarticular (n = 53), oligoarticular (n = 8), palindromic (n = 5) or polymyalgic (n = 6) disease. The cohort included 43 women, 24- to 75-years old (mean 49.1 years) and 29 men, 22- to 72-years old (mean 53.5 years). The erosion status was assessed from 24 to 74 months after the diagnosis according to the method of Larsen *et al*. [20]. Sera from 72 age and sex matched laboratory staff, non-RA hip surgery patients and medical students were used as controls. Positivity in anti-CCP or in anti-mutated citrullinated vimentin (MCV) assays was used as an excluding factor for the control population. The control sera were collected during 2010 to 2013. All sera were kept at −20°C. Preliminary testing of 38 hemodialysis patients was also performed to exclude the possible effect of high serum urea levels in antibody development (data not shown).

### Collagen telopeptide assays

All sera were tested by enzyme-linked immunosorbent assay (ELISA) for binding of immunoglobulin G (IgG) to citrullinated and homocitrullinated type I and II collagen carboxyterminal telopeptides under standard conditions, and the binding was inhibited by addition of soluble antigens [21] to a final concentration of 100 μg/ml. The two biotinylated peptides corresponding to the carboxyterminal telopeptide of the α1 chain of human type I collagen (NeoMPS, Strasbourg, France) represent amino acids 1193 to 1218, and the two biotinylated peptides corresponding to the α 1 chain of human type II collagen represent amino acids 1217 to 1241, counted from the amino-terminus in the pro α 1 chains of type I or type II procollagen, respectively. The synthetic peptides that were used as inhibitors in the ELISA represent the carboxyteminal ends of these parent peptides. The peptide sequences are given in Table 1. In each serum, the difference between bindings with and without inhibition was calculated for all peptide pairs. The patient results were divided into positive and negative using cut-off values defined as mean + 2 standard deviations (SD) of the control group (Table 2).

**Table 1:** **Synthetic peptides (SP) used in this study**

**Type I collagen**		**Sequence**	**Abbreviation**
SP112	biotin	SAG FDF SFL PQP PQE KAH DGG RYY **Cit** A	CitI
SP132	biotin	SAG FDF SFL PQP PQE KAH DGG RYY **Hcit** A	HcitI
SP66		-- --- --- --- --E KAH DGG RYY **Cit** A	CitI
SP135		-- --- --- --- --E KAH DGG RYY **Hcit** A	HcitI
**Type II collagen**			
SP86	biotin	GID MSA FAG LGP REK GPD PLQ YM **Cit** A	CitII
SP109	biotin	GID MSA FAG LGP REK GPD PLQ YM **Hcit** A	HcitII
SP41		--- --- --- --- -EK GPD PLQ YM **Cit** A	CitII
SP136		--- --- --- --- -EK GPD PLQ YM **Hcit** A	HcitII

The peptides represent different parts of the carboxy-terminal telopeptide of the α1 chain of human type I or II collagen, each containing citrulline (Cit) or homocitrulline (Hcit) as indicated (for other amino acids, the one-letter symbols are used).

**Table 2:** **Sensitivity and specificity of citrulline and homocitrulline inhibition-ELISA assays for separating RA patients from healthy controls**

**Test**	**Sensitivity**	**Specificity**
Anti-CCP	0.54	1.00^a^
Anti-MCV	0.57	1.00^a^
CitI	0.47	0.96
HcitI	0.22	0.96
CitII	0.41	0.94
HcitII	0.19	0.96

^a^Positivity in CCP or MCV was considered as excluding factor in controls. Anti-CCP, anti-cyclic citrullinated protein; anti-MCV, anti-mutated citrullinated vimentin; Cit, citrulline; Hcit, homocitrulline.

### Anti-CCP and anti-MCV assays

Anti-CCP (Immunoscan RA (Mark 2) from Euro Diagnostica, Malmö, Sweden) and anti-MCV (ORG 546, from ORGANTEC Diagnostica GmbH, Mainz, Germany) assays were performed according to the manufacturers’ instructions. All control and patient sera were tested in duplicate.

### Statistical methods

All 72 control sera included were negative for anti-CCP (<25 U/ml) and anti-MCV (<20 U/ml). Logistic regression analysis was used for evaluation of the association of each test or test type (citrulline or homocitrulline tests) with bone erosions in RA patients (IBM Corp. Released 2010. IBM SPSS Statistics for Windows, Version 19.0. Armonk, NY, USA). Differences were considered significant at *P* <0.01.

## Results

**Figure 1:**
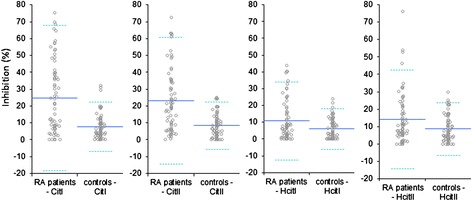
**Illustration of the specific binding (inhibition-%) in RA patients (n = 72) and in controls (n = 72).** Mean is shown as a solid line and ± 2SD is shown by dotted lines. For abbreviations, see Table 1. RA, rheumatoid arthritis; SD, standard deviation.

**Figure 2:**
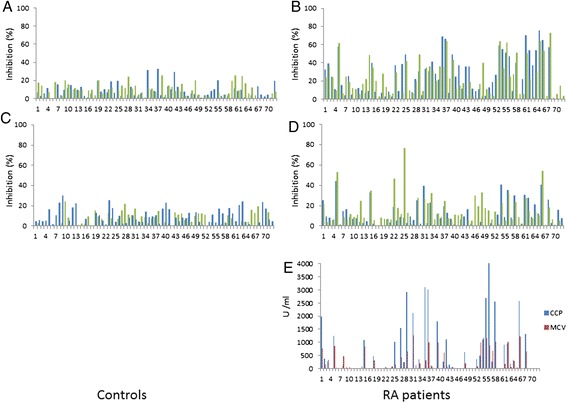
**Overview of the antibody specifities in controls (panels A and C) and RA patients (panels B and D) and RA patients’ CCP and MCV results.** In panels **A** and **B**, antibodies specific to CitI are depicted in dark columns/blue and those specific for HcitI in light columns/green. In panels **C** and **D**, antibodies to CitII are presented in dark columns/blue and those to HcitII in light column/green. The values shown are percent of inhibition by respective soluble peptides to binding to the similar immobilized peptide. Panel **E** shows the binding of RA patient sera to the antigens of the CCP and MCV assays, shown as arbitrary units defined by each manufacturer. CCP, cyclic citrullinated protein; Cit, citrulline; Hcit, homocitrulline; MCV, mutated citrullinated vimentin; RA, rheumatoid arthritis.

Patients with RA were found to have antibodies that bind to citrulline and homocitrulline-containing sequences related to type I and II collagen telopeptides (Figures 1 and 2). These antibodies were rare in healthy individuals (4% to 5% of the controls) and in hemodialysis patients (data not shown). The differences between patients and controls were statistically significant for all assays (Table 3).

**Table 3:** **Logistic regression analysis of the association of different antibody detection methods to erosions in RA patients**

**Test**	**OR**	**95% CI**	***P***
Anti-CCP	8.455	2.82 to 25.34	<0.001
Anti-MCV	15.583	4.76 to 51.05	<0.001
RF	7.506	2.54 to 22.20	<0.001
CitI	2.815	1.03 to 7.66	0.043
CitII	3.632	1.27 to 10.37	0.016
CitI or CitII	3.600	1.32 to 9.83	0.012
HcitI	2.400	0.69 to 8.40	0.171
HcitII	5.200	1.06 to 25.41	0.042
HcitI or Hcit II	3.692	1.08 to 12.61	0.037

CCP, cyclic citrullinated protein; CI, confidence interval; Cit, citrulline; Hcit, homocitrulline; MCV, mutated citrullinated vimentin; OR, odds ratio; RA, rheumatoid arthritis; RF, rheumatoid factor.

We measured specific antibodies to citrullinated type I collagen-related peptides using inhibition ELISA, where similar peptides were used both for binding the serum antibodies and in smaller, soluble form also to inhibit the binding. We found CitI specific antibodies in 34 (47%) of the 72 patient sera, while 16 (22%), 30 (42%) and 14 (19%) of the sera contained antibodies specific for HcitI, CitII and HcitII sequences, respectively. Furthermore, a total of 39 (54%) and 41 (57%) of the patient sera bound the respective antigens in direct ELISA for anti-CCP and anti-MCV. Total ELISA data can be found in Additional file 1.

Most of the sera that bound citrullinated peptides were reactive in multiple assays. A total of 10 (14%) sera contained specific antibodies to all four peptide pairs. Moreover, 28 (39%) of the patients had antibodies with overlapping specificities, so that the binding could be inhibited by peptides with different ureido group-containing amino acids. Of these, 16 (22%) sera showed such binding to citrulline-containing peptide that could be inhibited by homocitrulline-containing peptide. For another 16 (22%) sera the binding to homocitrulline-containing peptide could be inhibited by the corresponding citrulline-containing peptide. In only four (6%) of the cases were the antibodies cross-reactive and could be inhibited simultaneously in both directions.

We could analyze sera taken at two different time points from 19 patients (data not shown). There was only slight variation in the titers of antibodies to specific collagen telopeptide sequences between the two samples. We also preliminarily tested sera from 38 hemodialysis patients for binding to citrulline- and homocitrulline-containing peptides to exclude a possible effect of high serum urea levels and found the results comparable to those of healthy controls.

### Logistic regression analysis

The association of the results of each inhibition-ELISA to erosive disease was assessed using logistic regression analysis (Table 3). MCV had the strongest association to the development of erosions with an odds ratio (OR) of 15.6. CCP also was well associated to erosions, with an OR of 8, but it gave no additional value when combined with MCV. As expected, rheumatoid factor (RF) was also well associated with erosions with an OR of 7. The presence of antibodies to the collagen-related antigens CitI (OR 2.8; *P* = 0.043), CitII (OR 3.6; *P* = 0.016) and HcitII (OR 5.2; *P* = 0.042) reached close to statistically significant association with erosive RA, whereas for antibodies against HcitI (OR 2.4; *P* = 0.171) the association did not reach statistical significance. When the presence of joint erosions was checked against the presence of antibodies to either CitI or CitII in patient sera, the OR was 3.6 (*P* = 0.012), comparable to that of the combined homocitrulline tests; if a patient serum contained either HcitI or HcitII antibodies, the OR for erosions was 3.7 (*P* = 0.037).

## Discussion

We have previously obtained results in rabbit experiments indicating that antibodies against citrullinated antigens can also be induced with homocitrulline-containing immunogens [17] and that homocitrulline can be present in human RA tissue together with citrulline [10]. In this study, we wanted to define properties of RA antibodies that bind to citrulline- and homocitrulline-containing epitopes.

The antibodies binding to citrullinated and carbamylated proteins are induced even years prior to clinical RA onset [2,22]. The development is characterized by epitope spreading between antigens and the fine-tuning of the specificity of these antibodies [3,14]. Furthermore, in established disease the antibody repertoire remains fairly stabile [23]. The same phenomenon was also seen regarding antibodies to citrullinated and homocitrulline-containing collagen telopeptides.

We found that RA patients had specific antibodies to the CitI, CitII, HcitI and HcitII antigens. The antibodies binding to citrulline- or homocitrulline-containing telopeptides could be detected separately or simultaneously in the same serum. Evidently the antibodies present in sera of RA patients were able to discern between citrulline and homocitrulline in the same peptide-sequence, although there were also some overlapping specificities. The overlapping specificities were shown as the sera were able to bind to a citrullinated antigen, but could be inhibited by a soluble homocitrullinated peptide or vice versa. This phenomenon was usually detected only in one direction in a particular serum sample, but in two of the sera there was a cross-reaction in both directions (Additional file 1, inhibition table of all patients).

As we analyzed the two different serum samples of the same patient, the presence of antibodies to homocitrulline-containing antigens was found to be quite a constant phenomenon, in line with that of ACPAs. Once anti-homocitrulline antibodies appear in the sera they remain detectable irrespective of the disease status. Between the two samples the specificities were mainly widening but some fluctuation was seen in a few patients. A weakness of our study is that all samples had been collected in early RA (within a year from diagnosis) and no definite conclusions about long term variation between antibody titer can be drawn. In any case, it is worth noticing that the level of antibodies against homocitrulline-containing antigens in the 38 hemodialysis patient sera was comparable with that of healthy controls (data not shown).

Shi and others have previously found 45% of RA patients to be positive for IgG antibodies that recognize carbamylated protein [24]. The antigen used in this study was carbamylated fetal calf serum, a mixture of several carbamylated proteins. In the same study [24] anti-carbamylated protein antibodies were found to be related to erosive disease in an anti-CCP negative subgroup of RA patients. In our study, in two of the 42 erosive RA patients, homocitrulline binding antibodies were detected alone. All other patients were also positive for citrulline binding antibodies and/or for RF. Recently, also, in a large cohort study [25] antibodies binding to carbamylated proteins were mainly detected in ACPA-positive RA patients. In the present study, we found that anti-MCV had the strongest association with the development of erosive disease, followed by CCP and RF. Our study agrees with the results obtained by Mathsson and coworkers that the anti-MCV test is better than the anti-CCP-test to predict erosive disease [26]. In our study, combining these two tests did not give any additional value for predicting erosions. The results are in line with those obtained by van der Linden *et al*. [27].

The structural homology of two amino acids is a challenge for the immune system and a predisposing factor for development of overlapping specificities. In our study, the homocitrulline-containing sequences have no match in the human body, but the results show us properties of the binding antibodies. It is most probable that the level of cross-reacting antibodies is dependent on sequence homology of the neighboring amino acids in the immunogen and by analyzing with unnatural sequences it is not possible to directly identify the real target of these antibodies.

Our study does not reveal the original immunogen for anti-citrulline or anti-homocitrulline antibodies. The homocitrulline-containing type I and II collagen carboxyterminal telopeptides used in this study cannot be original immunogens, because the sequences we used do not exist in the body. However, the fact that homocitrulline-binding antibodies mainly exist in individuals showing binding in anti-CCP and anti-MCV assays suggests that some relationship between anti-homocitrulline and anti-citrulline antibodies exist.

At least one situation is known where citrullination and carbamylation are present together. NETs contain both hypercitrullinated histones and myeloperoxidase. In addition, in smokers the thiocynate released from tobacco smoke favors carbamylation. Thus, NETs in the oral cavity or in synovial tissue may be the place for the coexistence citrullination and carbamylation where the antibodies with overlapping specificities could also have a role in the development of chronic inflammation.

## Conclusions

Antibodies to both citrulline- and homocitrulline-containing sequences related to type I and II collagen telopeptides can be found in sera of RA patients. These antibodies are not constant from one RA patient to another, but contain separate or overlapping specificities within the same peptide sequence varying between individuals. Our results suggest a relationship between anti-citrulline and anti-homocitrulline antibodies, since anti-homocitrulline antibodies were found to exist mainly in individuals seropositive to anti-CCP and anti-MCV.

## Additional file

Additional file 1:
**Patient data.** This file contains RA patients’ primary Anti-CCP and Anti-MCV-ELISA results and inhibition results of the collagen telopeptide assays.

## Abbreviations

ACPA: anti-citrullinated protein antibodies
CCP: cyclic citrullinated protein
ELISA: enzyme-linked immunosorbent assay
IgG: immunoglobulin G
MCV: mutated citrullinated vimentin
NETs: neutrophil extracellular traps
RA: rheumatoid arthritis
RF: rheumatoid factor
