# Supplementary material for: Separate and overlapping specificities in rheumatoid arthritis antibodies binding to citrulline- and homocitrulline-containing peptides related to type I and II collagen telopeptides
Source: Arthritis Res Ther. 2015 Jan 9;17(1):2. doi: 10.1186/s13075-014-0515-z (PMC4320812; doi:10.1186/s13075-014-0515-z)
Supplement: Additional file 1: — Patient data. This file contains RA patients’ primary Anti-CCP and Anti-MCV-ELISA results and inhibition results of the collagen telopeptide assays. [file 13075_2014_515_MOESM1_ESM.pdf]

	anti-CCP	anti-MCV	CitI+CitI	CitI+HcitI	HcitI+CitI	HcitI+HcitI	CitII+CitII	CitII+HcitII	HcitII+CitII	HcitII+HcitII	erosion	RF	gender
1	+	+	+	-	-	+	+	-	-	-	+/-	+	f
2	+	+	+	-	-	-	+	-	-	-	+	+	m
3	+	+	+	-	+	-	+	-	-	-	+	+	f
4	-	-	-	-	-	-	-	-	-	-	-	-	f
5	+	+	+	-	-	+	+	-	-	+	-	+	m
6	+	-	-	-	-	-	-	-	-	-	+	+	f
7	-	-	-	+	-	-	+	+	+	-	-	-	f
8	+	+	+	+	-	-	-	-	-	-	+	+	f
9	-	+	-	-	-	-	-	-	-	-	+	-	f
10	-	+	-	-	-	-	-	-	-	-	+	-	f
11	-	-	-	-	-	-	-	-	-	-	+	+	f
12	-	-	-	-	-	-	-	-	-	+	+/-	+/-	f
13	-	-	-	-	-	-	-	-	-	-	-	-	m
14	-	+	-	-	-	-	+	-	+	-	+/-	+	m
15	+	+	+	-	-	+	+	-	+	+	+/-	+	f
16	-	-	-	-	-	-	-	-	-	-	-	-	f
17	-	-	-	-	-	-	-	-	-	-	-	+/-	f
18	+	+	-	-	-	-	+	-	-	-	+	+	f
19	-	-	-	-	-	-	-	-	-	-			f
20	-	-	-	-	-	-	-	-	-	-	-	-	f
21	-	-	-	+	-	-	-	-	-	-	+/-	-	m
22	+	+	+	+	+	+	+	+	-	+	+	+	m
23	-	-	-	-	-	-	-	-	-	-	-	-	f
24	+	+	+	-	-	-	-	-	-	-	-	+	f
25	+	+	+	+	-	-	+	+	-	+	+	+	f
26	-	-	-	-	-	-	-	-	-	-	-	-	m
27	+	+	-	-	-	-	-	-	-	-	+	+	m
28	+	+	-	-	-	-	-	-	-	-	+	+	f
29	+	+	+	-	+	+	+	-	-	+	+	-	m
30	-	-	-	-	-	-	-	-	-	-	-	-	f
31	+	+	+	-	-	+	+	+	-	+	+	+	f
32	+	-	+	-	-	-	+	+	-	-	-	+/-	f
33	+	+	+	-	-	+	+	-	-	+	-	+	m
34	-	-	+	+	-	-	-	+	-	-	-	-	f
35	+	+	-	+	-	-	+	+	-	-	+	+	f
36	+	+	+	-	-	-	+	+	-	-	+	+/-	f
37	+	+	+	-	-	+	+	-	-	+	+	+	f
38	-	-	-	-	-	-	-	-	-	-	+	+	f
39	+	+	+	-	+	-	+	-	-	-	-	+	m
40	-	-	+	-	-	-	-	-	-	-	-	-	f
41	+	+	+	-	-	-	-	-	-	-	+	+	m
42	+	+	-	-	+	-	-	-	-	-	+	+	f
43	+	+	+	-	-	-	-	-	-	-	+	-	m
44	+	+	+	-	+	-	-	-	-	-			f
45	-	-	-	-	-	-	-	-	-	-	-	-	m
46	-	-	-	-	-	-	-	-	-	+	+	-	f
47	-	-	-	-	-	-	-	+	-	-	+/-	-	f
48	+	+	-	-	-	-	+	-	-	+	+	+	m
49	-	-	-	-	-	-	-	-	-	-	-	-	m
50	-	-	-	+	-	-	-	-	-	-	-	-	f
51	-	-	-	-	-	-	-	-	-	-	-	-	m
52	+	+	+	-	-	-	-	+	-	-	+	+	f
53	+	+	+	-	-	-	+	-	+	-	+	+	m
54	+	+	+	-	-	+	+	-	-	-	-	-	m
55	+	+	+	-	-	-	+	-	-	-	+	+	m
56	+	+	+	-	-	+	+	-	-	+	+	-	f
57	+	+	-	-	-	-	+	-	-	-	+		f
58	+	+	+	-	-	+	+	-	-	-	+	-	m
59	-	-	-	-	-	-	-	-	-	-	-	-	f
60	-	-	-	-	-	-	-	-	-	-	+	+	f
61	+	+	+	-	+	+	+	-	-	+	+/-	+	m
62	+	+	+	+	+	+	-	-	-	-	+/-	+	f
63	+	+	+	-	+	-	-	-	-	-	+	+	m
64	+	+	+	-	-	+	+	-	-	-	+	+	f
65	-	+	+	-	+	-	+	+	-	-	+/-	+	m
66	+	+	+	-	-	+	+	-	-	+	+	+	m
67	-	-	-	-	-	-	-	-	-	-	-	-	m
68	+	+	+	-	-	+	+	-	+	-	-	+/-	m
69	-	-	-	-	-	-	-	-	+	-	-	-	f
70	-	-	-	+	-	-	-	-	-	-	-	+/-	m
71	-	-	-	-	-	-	-	-	-	-	-	-	f
72	-	-	-	-	-	-	-	-	-	-	-	+	m

Additional patient data. Anti-CCP and anti-MCV results are from direct ELISA, positivity was defined by the calibration series supplied by the manufacturer. For the other assays, results from inhibition-ELISAs are shown. Positivity was defined by inhibition values exceeding the mean + 2SD of controls. Erosions and RF were categorized as +, - or +/-. The borderline cases were defined as positive in logistic regression analysis. Gender is shown by one letter abbreviations: m, male and f, female. For the abbreviations of collagen telopeptide antigens, see Table 1.
